# Eco-friendly mixed metal (Mg–Ni) ferrite nanosheets for efficient electrocatalytic water splitting

**DOI:** 10.1038/s41598-023-49259-y

**Published:** 2023-12-13

**Authors:** Nyemaga M. Malima, Malik Dilshad Khan, Siphamandla C. Masikane, Felipe M. de Souza, Jonghyun Choi, Ram K. Gupta, Neerish Revaprasadu

**Affiliations:** 1 Department of Chemistry, University of Zululand, Private Bag X1001, KwaDlangezwa, 3880 South Africa; 2 Department of Chemistry, College of Natural and Mathematical Sciences, University of Dodoma, P.O. Box 338, Dodoma, Tanzania; 3 Department of Chemistry, National Institute for Materials Advancement, Pittsburg State University, Pittsburg, KS 66762 USA

**Keywords:** Electrochemistry, Inorganic chemistry, Materials chemistry

## Abstract

Eco-friendly and cost-effective catalysts with multiple active sites, large surface area, high stability and catalytic activity are highly desired for efficient water splitting as a sustainable green energy source. Within this line, a facile synthetic approach based on solventless thermolysis was employed for the simple and tunable synthesis of Ni_1−x_Mg_x_Fe_2_O_4_ (0 ≤ x ≤ 1) nanosheets. The characterization of nanosheets (via p-XRD, EDX, SEM, TEM, HRTEM, and SAED) revealed that the pristine ferrites (NiFe_2_O_4_ and MgFe_2_O_4_), and their solid solutions maintain the same cubic symmetry throughout the composition regulation. Elucidation of the electrochemical performance of the nanoferrite solid solutions showed that by tuning the local chemical environment of Ni in NiFe_2_O_4_ via Mg substitution, the intrinsic catalytic activity was enhanced. Evidently, the optimized Ni_0.4_Mg_0.6_Fe_2_O_4_ catalyst showed drastically enhanced HER activity with a much lower overpotential of 121 mV compared to the pristine NiFe_2_O_4_ catalyst. Moreover, Ni_0.2_Mg_0.8_Fe_2_O_4_ catalyst exhibited the best OER performance with a low overpotential of 284 mV at 10 mA/cm^2^ in 1 M KOH. This enhanced electrocatalytic activity could be due to improved electronic conductivity caused by the partial substitution of Ni^2+^ by Mg^2+^ in the NiFe_2_O_4_ matrix as well as the synergistic effect in the Mg-substituted NiFe_2_O_4_. Our results suggest a feasible route for developing earth-abundant metal oxide-based electrocatalysts for future water electrolysis applications.

## Introduction

The concerns related to the depletion of fossil fuels and the environmental pollution caused by their consumption call for an immediate search for alternate green and sustainable energy sources^1,2^. In this regard, hydrogen is a promising fuel with certain superiorities, such as high energy density, natural abundance and no harmful products created after burning^3–6^. Hydrogen generation through electrocatalytic water splitting is one of the most effective strategies, as water is cheap and abundant and hydrogen can be produced with high purity^6–8^. Water is thermodynamically highly stable and suitable electrocatalysts are required for water splitting. So far, noble metals-based electrocatalysts, such as IrO_2_/RuO_2_ for OER and Pt for HER, are benchmark catalysts for water splitting in terms of performance and stability, however, their high cost and scarcity are serious issues^9,10^. Efforts have been devoted to the exploration of suitable electrocatalysts with low overpotential, fast kinetic, and good stability for both hydrogen and oxygen evolution reactions (HER/OER) as well as overall water splitting^11,12^. For instance, various metal oxides^8,13,14^, sulfides^15,16^, selenides^17,18^, phosphides^19,20^, carbides and carbon nitride composites^8,21^, have been investigated as electrocatalysts. However, most of the non-oxide materials are unstable and convert to oxides or oxy-hydroxides during OER^22,23^. In this case, metal oxides can be used as stable bifunctional electrocatalysts but their activity is relatively low.

Some important pre-requisites for developing promising catalysts are that the catalyst must be composed of earth-abundant, eco-friendly and cost-effective materials. In addition, they should show high electrocatalytic activity and stability under catalytic conditions. Various reports suggest that multi-metal oxides are remarkably better than simple binary oxides^24,25^. The intrinsic activity can be tuned by tailoring the composition of multi-metal oxides. The activity enhancement occurs due to the creation of multiple active sites, which are critical for the rational design of superior electrocatalysts. In particular, creating atomic-scale synergistic active sites in single-phase systems is highly desirable.

Based on these principles, spinel ferrites can be earth-abundant, inexpensive, eco-compatible and highly effective materials, because of their sustainability under harsh conditions, high redox features, easy modulation in valence states, and enhanced electrical conductivity^26–28^. In particular, spinel NiFe_2_O_4_ and MgFe_2_O_4_ have gained recent attention and are being explored for possible use in energy storage and conversion technologies^29–34^. The two spinels crystalize in a cubic crystal symmetry and possess cations with variable oxidation states. These features enable the formation of a vast array of materials with tailored properties and promising electrochemical behavior^35–37^. To improve the electrocatalytic properties of NiFe_2_O_4_ and MgFe_2_O_4_, various strategies such as the formation of hybrid materials, doping, and interphase engineering have been employed. For instance, the Co-doped NiFe_2_O_4_ electrocatalyst with optimized composition (Co_6.25_Fe_18.75_Ni_75_O_*x*_) showed excellent OER activity with a small overpotential of 186 mV and a low Tafel slope of 38.5 mV/dec at 10 mA/cm^2^ in 1 M KOH^38^. A tailored interface engineering strategy has also been reported to enhance the electrocatalytic activity of NiFe_2_O_4_/NiTe for advanced energy conversion applications^39^. Diverse hybrid materials of NiFe_2_O_4_ or MgFe_2_O_4_ and graphene^40,41^, carbon nanotubes^42^, and other systems have also been investigated as potential candidates for electrocatalytic applications.

Although significant efforts have been devoted to improve the electrochemical properties of NiFe_2_O_4_ and MgFe_2_O_4_, there is a lack of study on the role of their substitutional solid solutions in electrocatalytic HER and OER. The formation of a solid solution allows for engineering the materials’ properties and performance via tunable composition^43^. Both NiFe_2_O_4_ and MgFe_2_O_4_ crystalize in a cubic symmetry and have comparable charges (+ 2) and crystal sizes of Ni^2+^ (0.69 Å) and Mg^2+^ (0.72 Å). This similarity allows the formation of a solid solution over an entire range of compositions and varying the stoichiometric amounts of Ni^2+^ and Mg^2+^ will result in the generation of multiple different active sites, a prerequisite for highly active catalysts.

Besides tailoring the composition, other ways of bolstering the performance are by engineering the morphology and avoiding the use of capping agents. The capping agents with long alkyl chains block the active sites and act as insulating layers, resulting in poor charge transfer properties^44^. Generally, the development of 2-dimensional morphology improves catalytic performance by providing enhanced surface area, which in turn results in more active sites. Likewise, the use of surfactants can be avoided by adopting solid-state pyrolysis of the precursors route. Therefore, this study reports a facile solvent-free approach to prepare nanosheets of inexpensive and eco-friendly Ni_1−x_Mg_x_Fe_2_O_4_ (0 ≤ x ≤ 1) solid solutions via pyrolysis of metal acetylacetonates. This straightforward and green synthesis procedure has afforded the formation of monophasic nanoferrite solid solutions that crystallize in a cubic spinel structure. The electrochemical performance of the nanoferrite solid solutions was investigated by tuning the local chemical environment of Ni in NiFe_2_O_4_ via Mg substitution.

## Experimental

### Chemicals

Nickel (II) acetylacetonate (98%, Merck-Schuchardt), magnesium (II) acetylacetonate (98%, Merck-Schuchardt), and iron (III) acetylacetonate (97%, Sigma-Aldrich). These metal complexes were used as received.

### Synthesis of Ni_1−x_Mg_x_Fe_2_O_4_ (0 ≤ x ≤ 1) solid solutions

The Ni_1−x_Mg_x_Fe_2_O_4_ (0 ≤ x ≤ 1) solid solutions of different stoichiometric compositions were prepared by solventless thermolysis of metal acetylacetonates. Briefly, for the synthesis of NiFe_2_O_4_ nanoparticles, 0.10 g (0.39 mmol) of nickel acetylacetonate and 0.27 g (0.78 mmol) of iron acetylacetonate were mixed to form a solid mixture. The solid mixture was ground using a pestle and mortar for ≈ 15 min to homogenize the mixture. The precursor mixture was then placed into a ceramic boat, which was placed in a reactor tube. The reactor tube was then placed inside the carbolite tube furnace, followed by thermal treatment at 450 °C, at a heating rate of 20 °C per minute for 1 h. After 1 h of annealing, the heating was switched off, and the furnace was allowed to cool to room temperature. The reactor tube was taken out of the furnace upon cooling, and the product was collected for analysis without any post-treatment. Similarly, the synthesis of MgFe_2_O_4_ nanoparticles was achieved by employing similar procedures except that magnesium acetylacetonate was used instead of nickel acetylacetonate and the amount of magnesium and iron complexes were maintained in the same mole ratio of 1:2.

For the synthesis of Ni_1−x_Mg_x_Fe_2_O_4_ (*x* = 0.2, 0.4, 0.6, 0.8) solid solutions, a known quantity of nickel acetylacetonate was partially substituted by appropriate amounts of magnesium acetylacetonate by adjusting the mole ratios of Mg and Ni in the intervals of 0.2, 0.4, 0.6, and 0.8, while keeping the amount of iron acetylacetonate unchanged in the reaction mixture. The reaction procedures for the entire series of solid solutions were kept similar to those employed to synthesize the ternary nickel and magnesium ferrites.

### Characterization of the Ni_1−x_Mg_x_Fe_2_O_4_ nanocatalysts

Structural analysis of the Ni_1−x_Mg_x_Fe_2_O_4_ nanoparticles was ascertained by powder X-ray diffraction (p-XRD) analysis employing a Bruker AXS D8 Advance X-ray diffractometer. The instrument uses nickel-filtered Cu Kα radiation (λ = 1.5418 Å) at 40 kV, 40 mA. SEM imaging was carried out on a ZEISS-Auriga Cobra SEM Field Emission Scanning Electron Microscope (FE SEM) while EDX elemental analysis was performed on a JEOL JSM-7500F Field Emission Scanning Electron Microscope (FE-SEM) equipped with Energy Dispersive X-ray spectroscopy (EDX). The SAED, TEM and HRTEM analyses were performed on a JEOL 2100 HRTEM at accelerating voltages of 200 kV.

### Electrochemical characterization

The electrocatalytic property of the Ni_1−x_Mg_x_Fe_2_O_4_ (0 ≤ x ≤ 1) was examined via a three-electrode system using a Versastat 4-500 electrochemical workstation (Princeton Applied Research, Oak Ridge, TN, USA). For the preparation of the working electrode, the electrode paste was synthesized using Ni_1−x_Mg_x_Fe_2_O_4_ material (80%), PVDF (10%), carbon black (10%) with *N*-methyl pyrrolidinone (NMP) solvent as active materials, binder, and conducting agent, respectively. The paste was dipped into the clean Ni foam and dried for 48 h. While Pt wire was used as a counter electrode, Hg/HgO was used as the reference electrode. To examine the performance of the electrocatalyst for HER and OER, linear sweep voltammetry (LSV) was carried out at a scan rate of 2 mV/s. Also, electrochemical impedance spectroscopy (EIS) was performed at the potential of 0.6 V (V, SCE) in the frequency range of 0.05 Hz–10 kHz at an applied AC amplitude of 10 mV. For the stability of electrocatalysts, chronoamperometry techniques were utilized at the potential of 0.57 V (V, SCE). All measurements for electrocatalysts were conducted using 1 M KOH electrolyte.

## Results and discussion

### Structural analysis of Ni_1−x_Mg_x_Fe_2_O_4_ (0 ≤ x ≤ 1) solid solutions

**Figure 1:**
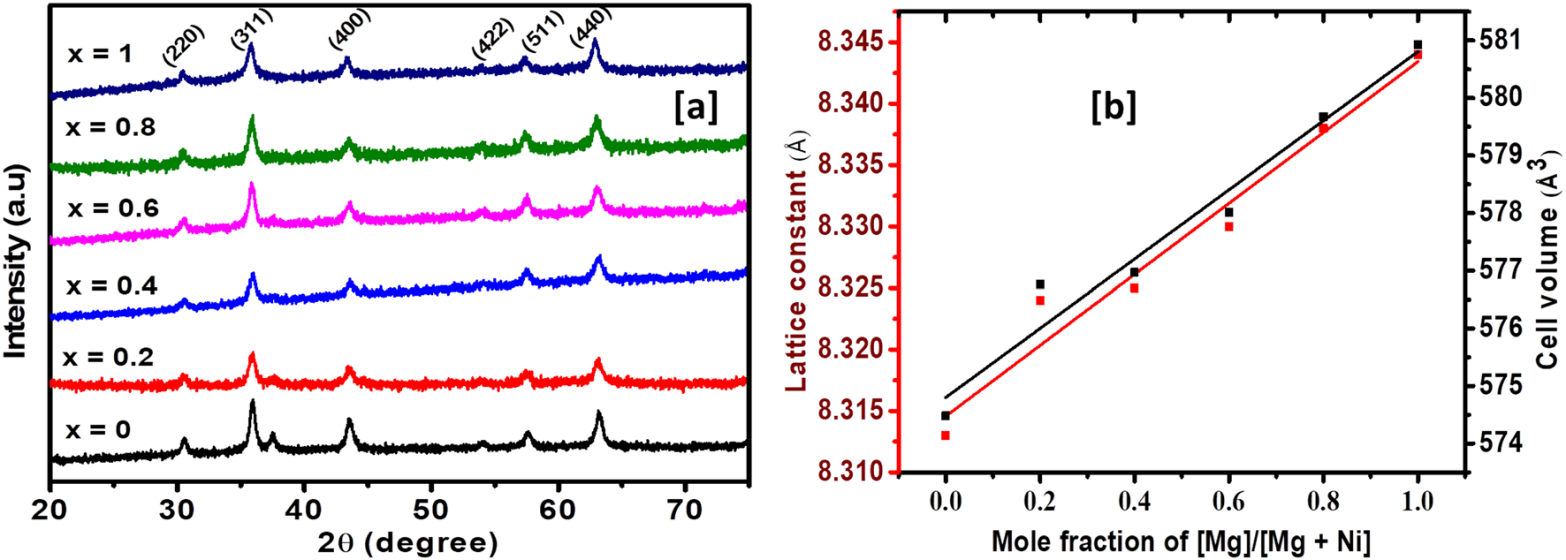
(**a**) Powder-XRD patterns (**b**) Variation of lattice constant (left y-axis) and cell volume (right y-axis) with Mg^2+^ content for Ni_1−x_Mg_x_Fe_2_O_4_ (0 ≤ x ≤ 1) solid solutions.

A set of Ni_1−x_Mg_x_Fe_2_O_4_ nanocatalysts were synthesized by a direct solid-state thermolysis process. Figure 1a shows the typical powder X-ray diffraction (p-XRD) patterns of the as-prepared Ni_1−x_Mg_x_Fe_2_O_4_. The diffraction peaks found in the pure ternary systems prepared at x = 0 and x = 1 are exclusively indexed with the cubic spinel crystal system having the space group *Fd*3̅*m*. The pristine ferrites are consistent with the cubic phases of pure trevorite, NiFe_2_O_4_ (ICDD # 01-086-2267) and magnesioferrite, MgFe_2_O_4_ (ICDD # 01-089-3084) for x = 0 and x = 1, respectively. The p-XRD data for the nanoferrites with x = 0.2 to x = 0.8 indicate the formation of solid solution phases with variable stoichiometric composition of Ni^2+^ and Mg^2+^ in the spinel matrix. Notably, their diffraction peaks lie in between those of pure NiFe_2_O_4_ and MgFe_2_O_4_, and these solid solutions maintained the same cubic symmetry throughout the composition regulation.

The lattice constants (*a* = *b* = *c*) of Ni_1−x_Mg_x_Fe_2_O_4_ (0 ≤ x ≤ 1) nanospinels were ascertained from the p-XRD data by employing the formula shown by Eq. (1) and the results are shown in Table 1.

1 $$\frac{1}{{d^{2} }} = \frac{{\left( {h^{2} + k^{2} + l^{2} } \right)}}{{a^{2} }}$$

**Table 1:** Lattice parameter (a), crystallite size (d), unit cell volume (V), and EDX composition of nanospinel Ni_1−x_Mg_x_Fe_2_O_4_ solid solutions at various magnesium contents (x).

(x)	Target ferrite composition	Stoichiometry obtained from EDX	a (Ȧ)	d (nm)	V (Ȧ^3^)
0	NiFe_2_0_4_	Ni_1.0_Fe_1.8_0_4.2_	8.313	19	574.478
0.2	Ni_0.8_Mg_0.2_Fe_2_O_4_	Ni_0.74_Mg_0.23_Fe_1.8_O_4.07_	8.324	17	576.761
0.4	Ni_0.6_Mg_0.4_Fe_2_O_4_	Ni_0.43_Mg_0.40_Fe_2.03_O_4.14_	8.325	10	576.969
0.6	Ni_0.4_Mg_0.6_Fe_2_O_4_	Ni_0.41_Mg_0.58_Fe_1.90_O_4.11_	8.330	13	578.010
0.8	Ni_0.2_Mg_0.8_Fe_2_O_4_	Ni_0.13_Mg_0.75_Fe_1.88_O_4.25_	8.338	14	579.676
1	MgFe_2_O_4_	Mg_0.99_Fe_1.83_O_4.18_	8.344	12	580.929

The lattice constants of NiFe_2_O_4_ were found to be 8.313 Å, conforming to those reported in the standard data (8.337 Å, ICDD #: 01-086-2267). After the incorporation of Mg^2+^, a slight increase in the values of the lattice parameters is observed, which is also ascribed to the slightly larger size of Mg^2+^ (0.72 Å) relative to the Ni^2+^ (0.69 Å)^45^. The lattice parameters computed for pure MgFe_2_O_4_ (8.344 Å) are also comparable with the values reported in standard data (8.369 Å, ICDD #: 01-089-3084). The values of lattice parameters were then plotted as a function of Mg^2+^ content (*x*) as shown in Fig. 1b. It is obvious that the lattice constant increases in a linear fashion with Mg^2+^ inclusion from 8.313 Å for NiFe_2_O_4_ to 8.344 Å for MgFe_2_O_4_. This linear relationship between the lattice parameters and Mg^2+^ content is in agreement with Vegard’s law^46^. The values of the lattice constants obtained in this study are consistent with previously reported values for magnesium-substituted nickel ferrite nanoparticles prepared via a co-precipitation route^47^. Likewise, the data in Table 1 and Fig. 1b demonstrate that the cell volume increases monotonically with increasing magnesium content. All these findings confirm the successful inclusion of Mg^2+^ into the crystal structure of NiFe_2_O_4_. The Debye–Scherrer formula (Eq. 2), Ref.^48^ was employed to compute the average crystallite size of Ni_1−x_Mg_x_Fe_2_O_4_ samples.

2 $$L = \frac{0.89\lambda }{{\beta cos\theta }}$$

In the formula, *L* = average crystallite size, λ = X-ray wavelength, *β* = full width at half maximum, and *θ* = Bragg’s angle of the (311) plane. The average crystallite sizes of the as-prepared Ni_1−x_Mg_x_Fe_2_O_4_ nanoparticles vary between 10 and 20 nm (Table 1). The average crystallite size obtained for the pristine nickel ferrite was larger compared to those exhibited by magnesium-substituted samples.

### Elemental compositional analysis

**Figure 2:**
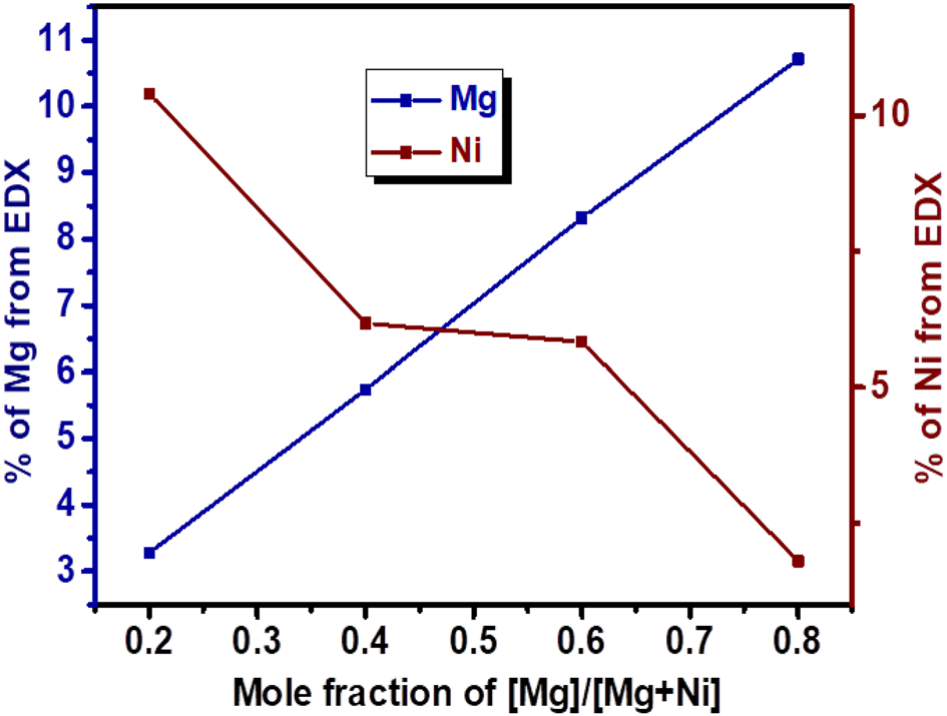
Change in Mg and Ni content as a function of mole fraction of [Mg]/[Mg + Ni] in precursor feed.

**Figure 3:**
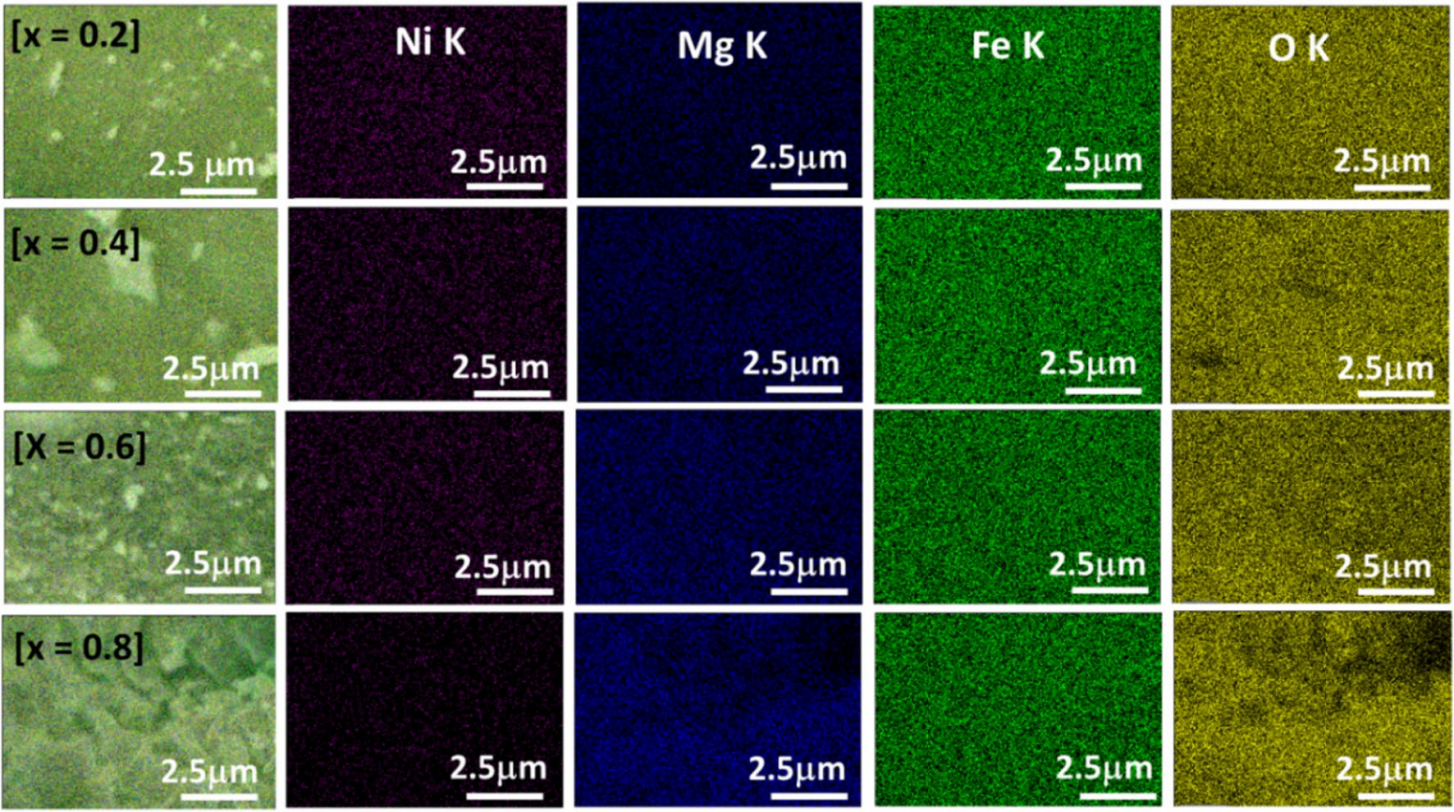
Elemental mapping of Ni_1−x_Mg_x_Fe_2_O_4_ (0.2 ≤ x ≤ 0.8) nanosheets.

The composition and elemental distributions of Ni, Mg, Fe, and O were analyzed by energy-dispersive X-ray (EDX). The EDX results (Supplementary Information, Fig. S1) indicate the presence of Ni, Fe and O for x = 0, and Mg, Fe and O for x = 1 in the desired ratio. For the solid solution nanocrystals with compositions x = 0.2–0.8, the presence of Ni, Mg, Fe, and O was confirmed. A summary of the atomistic composition of each of the components in the alloyed nanoferrites is provided in Table S1. The stoichiometry of the elements obtained is consistent with the expected values within the substitution limits, suggesting that there is no side reaction or significant loss of the starting materials. In Fig. 2, the relationship between the amount of Ni^2+^ and Mg^2+^ detected from EDX with respect to the mole fraction of [Mg]/[Mg + Ni] in precursor feed indicates a decrease in nickel content with a linear increase in magnesium content. In addition, the EDX mapping of the as-prepared Ni_1−x_Mg_x_Fe_2_O_4_ solid solutions is given in Fig. 3, indicating that the distribution of the respective elements in the spinel structure is nearly uniform, ruling out the possibility of de-alloying or phase segregation. This also confirms the formation of the solid solution between NiFe_2_O_4_ and MgFe_2_O_4_ in the single-crystalline alloyed nanospinel.

### Microstructure and morphological studies

**Figure 4:**
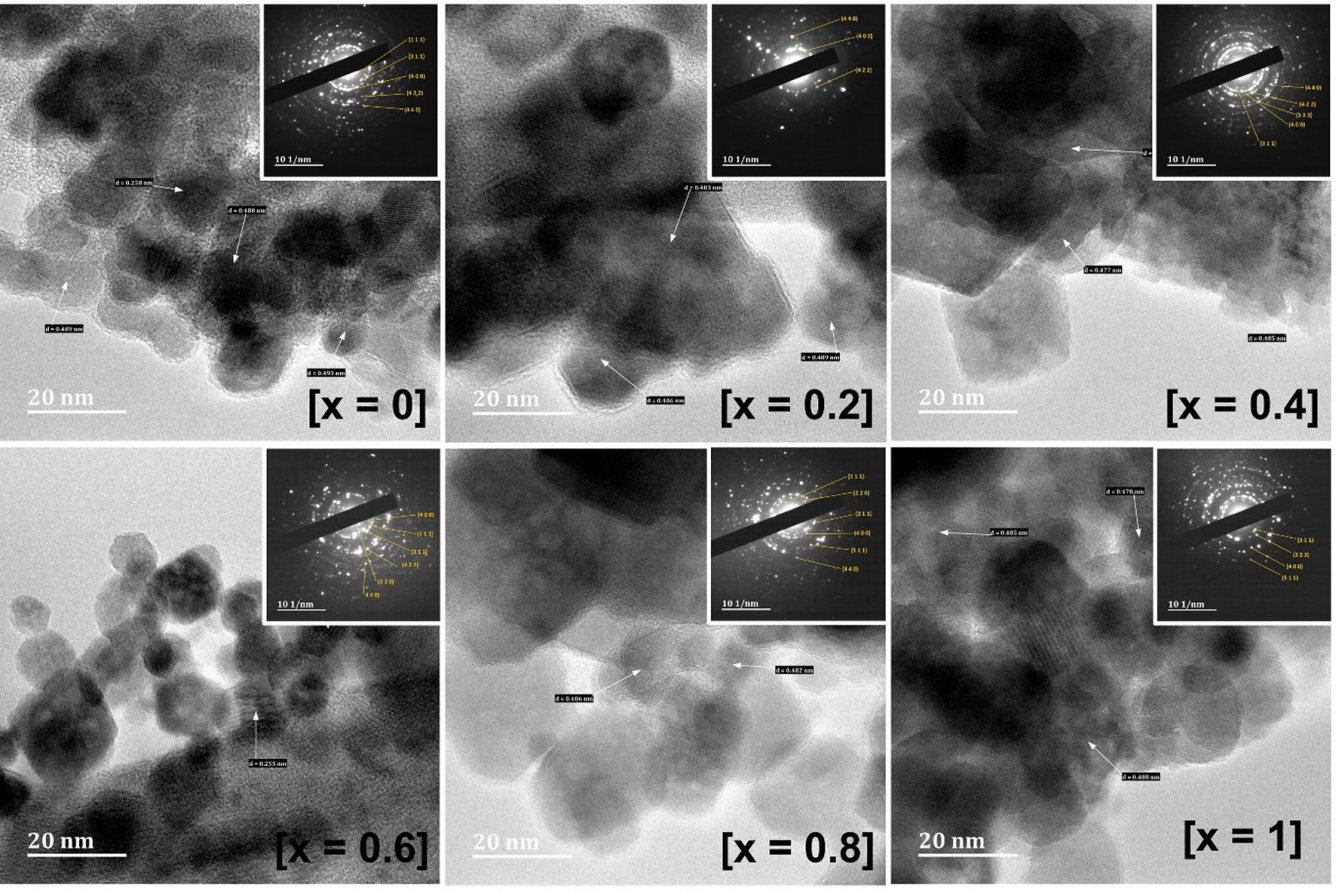
TEM and SAED (insets) images of Ni_1−x_Mg_x_Fe_2_O_4_ solid solutions over the entire range, by solid-state decomposition of metal acetylacetonate complexes.

The morphology of nanoparticulate Ni_1−x_Mg_x_Fe_2_O_4_ (0 ≤ x ≤ 1) solid solutions was examined by using SEM and TEM analyses. Figure S2 shows SEM micrographs of Ni_1−x_Mg_x_Fe_2_O_4_ nanoparticles. It can be seen that for all compositions, nanoclusters were formed with a narrow size distribution, and a closer look indicates that the clusters are composed of smaller particles grouped together. To have clear information about the particle morphology, size and microstructure of Ni_1−x_Mg_x_Fe_2_O_4_ samples, TEM analysis was carried out. The TEM images displayed in Fig. 4 show sheet-like structures with truncated edges. The nanosheets were of different sizes in the range of 10–100 nm, where smaller particles coalesced together to form relatively bigger sheets. The TEM analysis also shows that the size of nanosheets was relatively small for NiFe_2_O_4_ and the size increases with increasing Mg concentration in Ni_1−x_Mg_x_Fe_2_O_4_. The nanosheets formed were of low thickness and some sheets were horizontally stacked on top of other sheets. Additionally, the lattice fringes with interplaner spacings of d = 4.81, 2.94, 2.51, 2.40, and 2.08 Å were observed, corresponding to the (111), (220), (311), (222), and (400) planes of cubic spinel Ni_1−x_Mg_x_Fe_2_O_4_ nanosheets. These results are consistent with the characteristic d-spacing and Miller indices observed from p-XRD data. Further, the SAED patterns (displayed as insets) reveal well-defined spots, which suggest the crystalline nature of Ni_1−x_Mg_x_Fe_2_O_4_ samples.

### Electrocatalytic HER and OER

**Figure 5:**
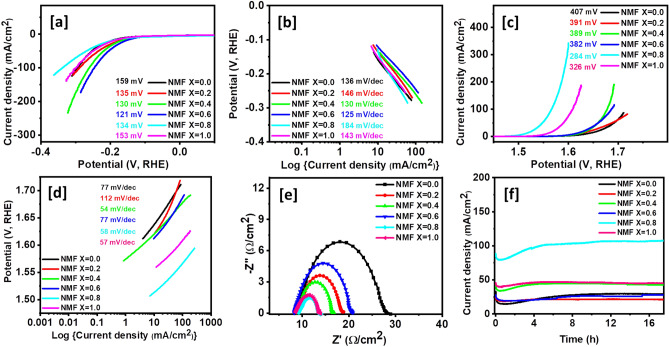
(**a**) HER polarization curves, (**b**) HER Tafel slopes, (**c**) OER polarization curves, (**d**) OER Tafel slopes, (**e**) Nyquist plots at 0.5 V, and (**f**) Chronoamperometric measurements at 0.55 V, for Ni_1−x_Mg_x_Fe_2_O_4_ (0 ≤ x ≤ 1) electrodes (NMF stands for Ni_1−x_Mg_x_Fe_2_O_4_ (0 ≤ x ≤ 1)).

For electrocatalytic analysis, Ni_1−x_Mg_x_Fe_2_O_4_ (0 ≤ x ≤ 1) will be referred to as NMF with a specified “x” value. The HER activities of Ni_1−x_Mg_x_Fe_2_O_4_ (0 ≤ x ≤ 1) catalysts were investigated under alkaline conditions (1 M KOH) in a usual three-electrode arrangement. Figure 5a displays the LSV polarization curves of Ni_1−x_Mg_x_Fe_2_O_4_ with varying mole ratios of Ni and Mg at a scan rate of 5 mV/s. Pure NiFe_2_O_4_ displays low catalytic performance with an overpotential of 159 mV which was needed to produce a current density of 10 mA/cm^2^. Upon Mg incorporation, the electrocatalytic activity was enormously improved as manifested by the reduction of overpotential from 159 mV of bare NiFe_2_O_4_ to Ni_0.8_Mg_0.2_Fe_2_O_4_ (135 mV), Ni_0.6_Mg_0.4_Fe_2_O_4_ (130 mV), Ni_0.4_Mg_0.6_Fe_2_O_4_ (121 mV), Ni_0.2_Mg_0.8_Fe_2_O_4_ (134 mV), and MgFe_2_O_4_ (153 mV). Remarkably, at the current density of 10 mA/cm^2^, the Ni_1−x_Mg_x_Fe_2_O_4_ (x = 0.6) electrode exhibited the best electrocatalytic activity for HER with an overpotential of 121 mV which is smaller compared to its counterparts. This reduction in overpotentials demonstrates that the incorporation of the proper content of Mg in the crystal lattice of NiFe_2_O_4_ can effectively improve its catalytic activity for HER. These results further demonstrated that the value of overpotential of Ni_0.4_Mg_0.6_Fe_2_O_4_ at the geometric current density of 10 mA/cm^2^ is superior to many binary and ternary metal oxide catalysts such as NiFe_2_O_4_ (290 mV)^49^, Ni/Co_3_O_4_ (145 mV)^50^, Fe_2_O_3_/NCs (350 mV)^51^, δ-MnO_2_ (196 mV)^52^, and MgFe_2_O_4_ (402 mV)^53^. It is noteworthy that the Ni_0.4_Mg_0.6_Fe_2_O_4_ also shows great superiority to other previously reported HER electrocatalysts summarized in Table S2. In Fig. 5b, the Tafel slopes of all electrodes were measured from the LSV measurements. Remarkably, the Tafel slope of Ni_0.4_Mg_0.6_Fe_2_O_4_ was found to be 125 mV/dec, which is lower than that of NiFe_2_O_4_ (136 mV/dec), Ni_0.8_Mg_0.2_Fe_2_O_4_ (146 mV/dec), Ni_0.6_Mg_0.4_Fe_2_O_4_ (130 mV/dec), Ni_0.8_Mg_0.2_Fe_2_O_4_ (184 mV/dec), and MgFe_2_O_4_ (143 mV/dec). The reduction of Tafel slope from 136 mV/dec (NiFe_2_O_4_) to 125 mV/dec (Ni_0.4_Mg_0.6_Fe_2_O_4_) may be ascribed to probable modification effect of the surface electronic state due to incorporation of Mg element, which in turn enhances the inherent conductivity of Ni_0.4_Mg_0.6_Fe_2_O_4_^54^. These changes indicate that among other factors, the electrochemical kinetics depend on the ratio of Mg dopants. Generally, the lower Tafel slope of the electrode indicates better process kinetics, even when significant H_2_ generation is needed at elevated voltage or current densities.

The OER activities of a series of Ni_1−x_Mg_x_Fe_2_O_4_ (0 ≤ x ≤ 1) catalysts in 1 M KOH solution were also investigated. The polarization curves of Ni_1−x_Mg_x_Fe_2_O_4_ catalysts with different mole ratios of Ni to Mg show a significant decrease in the overpotential upon Mg incorporation in NiFe_2_O_4_ (Fig. 5c). While the pristine NiFe_2_O_4_ needed an overpotential of 407 mV to deliver a current density of 10 mA/cm^2^, the Mg-doped NiFe_2_O_4_ samples exhibited lower overpotentials of 391, 389, 382, and 284 mV for x = 0.2, 0.4, 0.6 and 0.8, respectively. Also, the pristine MgFe_2_O_4_ displayed a lower overpotential of 326 mV compared to NiFe_2_O_4_. Among the studied series of Ni_1−x_Mg_x_Fe_2_O_4_ (0 ≤ x ≤ 1) catalysts, Ni_0.2_Mg_0.8_Fe_2_O_4_ demonstrated the best OER performance with a lower overpotential of 284 mV, within the window of potential examined. The electrocatalytic activity demonstrated by Ni_0.2_Mg_0.8_Fe_2_O_4_ surpasses many other previously reported metal oxide-based catalysts. For example, MnFe_2_O_4_ was reportedly synthesized by Li et al. and showed an overpotential of 470 mV at a current density of 10 mA/cm^2^ in alkaline media^55^. In a similar study, CoFe_2_O_4_ exhibited 370 mV under similar electrolytic conditions. Hirai et al. reported that Mn_3_O_4_ needed an overpotential of 600 mV to produce a current density of 10 mA/cm^2^ in 1 M KOH solution. They further reported the synthesis of Mn_2.4_Co_0.6_O_4_ which exhibited a high overpotential of 510 mV^56^. Also, Co_3_O_4_ nanocubes fabricated by Chen et al. were reported to display an overpotential of 580 mV (at 10 mA/cm^2^) in alkaline electrolytes^57^. Table S3 shows the comparison of the values of overpotentials Ni_0.2_Mg_0.8_Fe_2_O_4_ with other non-precious metal catalysts. The values of the Tafel slope indicated in Fig. 5d were obtained in the range of 54–112 mV/dec. The low overpotential and small Tafel slope make Ni_0.2_Mg_0.8_Fe_2_O_4_ a more promising OER catalyst.

The electrical conductivity of Ni_1−x_Mg_x_Fe_2_O_4_ (0 ≤ x ≤ 1) nanocatalyts was elucidated by electrochemical impedance spectroscopy. The Nyquist plot displayed in Fig. 5e shows that the pristine NiFe_2_O_4_ nanoparticles possess a large semicircle, demonstrating poor electron transfer capability, compared to Ni_1−x_Mg_x_Fe_2_O_4_ (0.2 ≤ x ≤ 0.8) solid solutions. The lowest charge resistance values displayed by the solid solutions imply intimate contact between the current collector and Ni_1−x_Mg_x_Fe_2_O_4_ and is an indication of more swift charge transfer kinetics. The results confirm further that the incorporation of Mg in spinel NiFe_2_O_4_ lattices contributed to the improvement of electrical conductivity via reduction of the charge transfer resistance, and consequently boosting the electrocatalytic properties of Ni_1−x_Mg_x_Fe_2_O_4_ electrodes. Of all electrode configurations investigated, Ni_0.2_Mg_0.8_Fe_2_O_4_ shows the smallest semicircle, indicating superior conductivity, and hence high electrocatalytic activity towards water splitting.

The diameter of the semicircle obtained at a lower frequency provides information on the ionic resistance of the electrolyte, indicating series resistance (R_s_) with charge transfer resistance R_ct_. The R_s_ for all the samples studied were the same, while R_ct_ depends on the composition. The simplest equivalent circuit for these samples is where one resistance R_s_ in series with C_dl_ and R_ct_ parallel to the C_dl_. The composition of Ni_1−x_Mg_x_Fe_2_O_4_ with X = 0 showed the highest charge-transfer resistance while with X = 0.8 displayed the lowest charge-transfer resistance.

**Figure 6:**
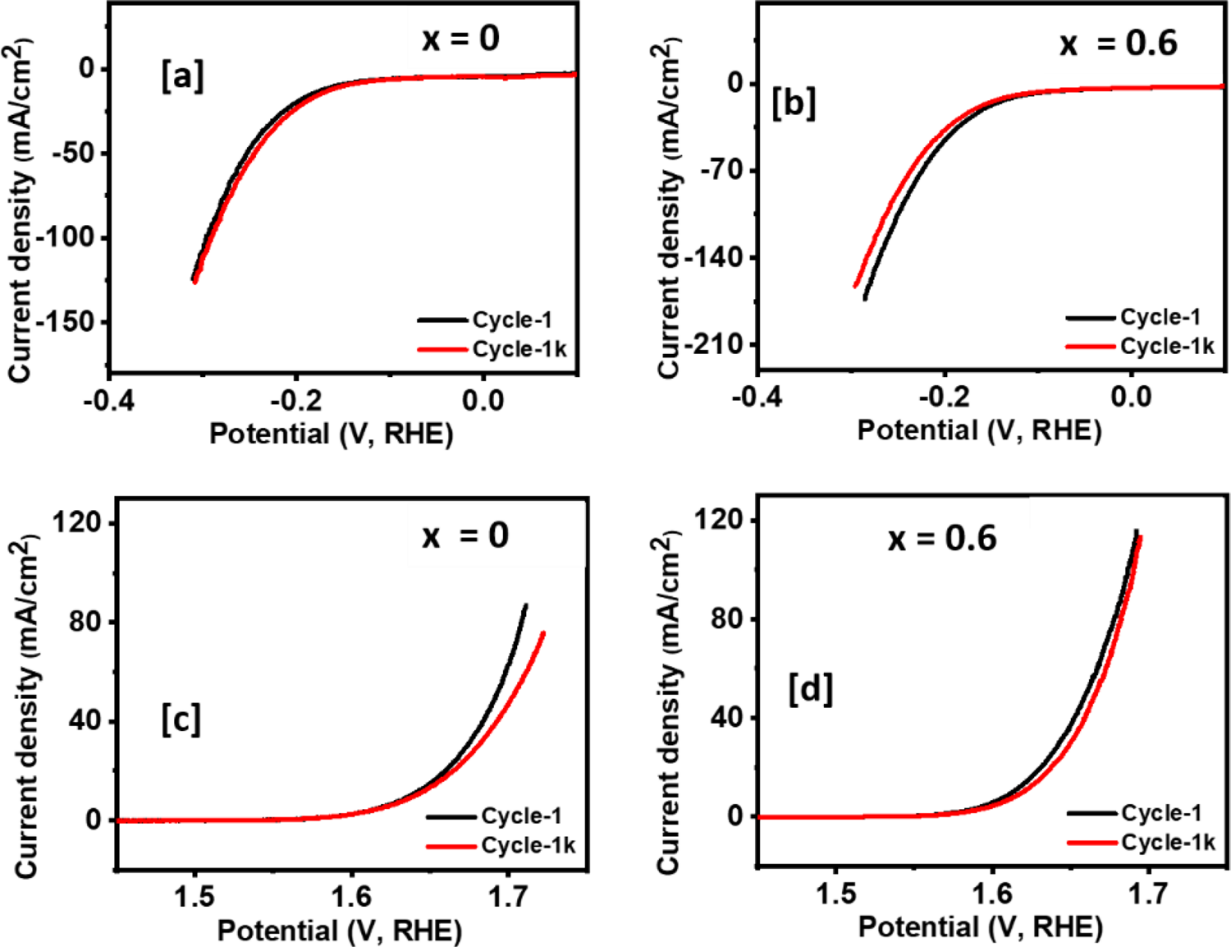
Comparison of HER (**a**, **b**) and OER (**c**, **d**) polarization curve between LSV 1 curve and LSV 1 k curve for Ni_1−x_Mg_x_Fe_2_O_4_ (x = 0 and 0.6) electrodes.

Evaluation of the catalyst’s electrochemical stability is important for practical water splitting applications. To explore the electrochemical stability of Ni_1−x_Mg_x_Fe_2_O_4_ (0 ≤ x ≤ 1) electrocatalysts, chronoamperometry measurements were performed at 0.55 V. Remarkably, no significant changes in the current density were observed during 17 h tests, signifying excellent electrochemical stability of all Ni_1−x_Mg_x_Fe_2_O_4_ systems in the alkaline electrolyte (Fig. 5f). The stability of Ni_1−x_Mg_x_Fe_2_O_4_ (0 ≤ x ≤ 1) electrocatalysts was further examined by continuous LSV scans in 1 M KOH. The results indicate the absence of significant change in the polarization curve after 1000 cycles, signifying superior stability of the nanocatalysts for both HER (Fig. 6a,b, and Fig. S3) and OER (Fig. 6c,d, and Fig. S4) in alkaline solution.

## Conclusion

In conclusion, this study reports a composition-controlled fabrication of homogeneous Ni_1−x_Mg_x_Fe_2_O_4_ (0 ≤ x ≤ 1) solid solutions by solventless pyrolysis method. Experimental investigation demonstrates that by regulating the molar composition of Mg and Ni in the preparation process, the physicochemical and electrochemical performance of the material were modified. The as-synthesized Ni_0.4_Mg_0.6_Fe_2_O_4_ nanoparticles exhibited the best electrocatalytic activity for HER with an overpotential of only 121 mV which is much smaller compared to its analogues, at a current density of 10 mA/cm^2^ and the electrode exhibits good stability during long-term electrolysis. Meanwhile, Ni_0.2_Mg_0.8_Fe_2_O_4_ showed the best OER activity, requiring an overpotential of 284 mV to deliver the same current density within the window of potential examined. The outstanding electrocatalytic performance of these solid solutions is largely ascribed to the enhanced conductivity due to surfactant free surfaces, nanoparticulate nature and synergic effect of different metals (Mg, Ni and Fe) which either directly or indirectly promoted the catalytic activity. The results described in this work pave the way for the design of mixed spinel oxides with high electrocatalytic activity for applications in sustainable energy systems.

### Supplementary Information

Supplementary Information.

## Data Availability

All data generated or analysed during this study are included in this published article and its supplementary information files. Further data is available from the corresponding author on reasonable request.

## References

[1] Bhowmik C, Bhowmik S, Ray A (2020). Optimal green energy source selection: An eclectic decision. Energy Environ..

[2] Baidya, S. & Nandi, C. Green energy generation using renewable energy technologies. *Adv. Greener Energy Technol*. **1**, 259–276 (2020).

[3] Li X (2023). Latest approaches on green hydrogen as a potential source of renewable energy towards sustainable energy: Spotlighting of recent innovations, challenges, and future insights. Fuel.

[4] Wang M, Wang G, Sun Z, Zhang Y, Xu D (2019). Review of renewable energy-based hydrogen production processes for sustainable energy innovation. Glob. Energy Interconnect..

[5] Mugheri AQ (2020). Electrospun fibrous active bimetallic electrocatalyst for hydrogen evolution. Int. J. Hydrogen Energy.

[6] Wang X (2018). In situ synthesis of hierarchical MoSe_2_–CoSe_2_ nanotubes as an efficient electrocatalyst for the hydrogen evolution reaction in both acidic and alkaline media. J. Mater. Chem. A.

[7] Sun L, Luo Q, Dai Z, Ma F (2021). Material libraries for electrocatalytic overall water splitting. Coordination Chem. Rev..

[8] Mugheri AQ, Otho AA, Mugheri AA (2021). Meritorious spatially on hierarchically Co_3_O_4_/MoS_2_ phase nanocomposite synergistically a high-efficient electrocatalyst for hydrogen evolution reaction performance: Recent advances & future perspectives. Int. J. Hydrogen Energy.

[9] Suen N-T (2017). Electrocatalysis for the oxygen evolution reaction: Recent development and future perspectives. Chem. Soc. Rev..

[10] Zou X, Zhang Y (2015). Noble metal-free hydrogen evolution catalysts for water splitting. Chem. Soc. Rev..

[11] Mugheri AQ, Otho AA, Abro MA, Ali A, Khan S (2021). Versatile noble-metal-free electrocatalyst synergistically accelerating for the highly comprehensive understanding evidence for electrochemical water splitting: Future achievements & perspectives. Surfaces Interfaces.

[12] Mugheri AQ, Samtio MS, Sangah AA, Awan JH, Memon SA (2021). Promoting highly dispersed Co_3_O_4_ nanoparticles onto polyethyne unraveling the catalytic mechanism with stable catalytic activity for oxygen evolution reaction: From fundamentals to applications. Int. J. Hydrogen Energy.

[13] Malima NM, Khan MD, Choi J, Gupta RK, Revaprasadu N (2023). Alloying normal and inverse spinel (Zn–Co ferrite) nanostructures via direct precursor pyrolysis for enhanced supercapacitance and water splitting. Mater. Chem. Phys..

[14] Malima NM (2021). Solventless synthesis of nanospinel Ni_1__−__x_Co_x_Fe_2_O_4_ (0 ≤ x ≤ 1) solid solutions for efficient electrochemical water splitting and supercapacitance. RSC Adv..

[15] Shombe GB (2020). Unusual doping induced phase transitions in NiS via solventless synthesis enabling superior bifunctional electrocatalytic activity. Sustain. Energy & Fuels.

[16] Khan, M. D., Warczak, M., Shombe, G. B., Revaprasadu, N. & Opallo, M. Molecular precursor routes for Ag-based metallic, intermetallic, and metal sulfide nanoparticles: Their comparative ORR activity trend at solid|liquid and liquid|liquid interfaces. *Inorg. Chem*. 8379–8388 (2023). 10.1021/acs.inorgchem.3c00978 PMC10230501 37191662

[17] Razzaque S (2021). Selective synthesis of bismuth or bismuth selenide nanosheets from a metal organic precursor: Investigation of their catalytic performance for water splitting. Inorg. Chem..

[18] Peng X (2020). Recent advance and prospectives of electrocatalysts based on transition metal selenides for efficient water splitting. Nano Energy.

[19] Ayom GE (2020). Flexible molecular precursors for selective decomposition to nickel sulfide or nickel phosphide for water splitting and supercapacitance. Chem. Eur. J..

[20] Wang Y, Kong B, Zhao D, Wang H, Selomulya C (2017). Strategies for developing transition metal phosphides as heterogeneous electrocatalysts for water splitting. Nano Today.

[21] El Rouby WM (2020). Synthesis and characterization of Bi-doped g-C_3_N_4_ for photoelectrochemical water oxidation. Solar Energy.

[22] Zuo Y (2019). In situ electrochemical oxidation of Cu_2_S into CuO nanowires as a durable and efficient electrocatalyst for oxygen evolution reaction. Chem. Mater..

[23] Wang T (2018). NiFe (Oxy) hydroxides derived from NiFe disulfides as an efficient oxygen evolution catalyst for rechargeable Zn–air batteries: The effect of surface S residues. Adv. Mater..

[24] Kim JS, Kim B, Kim H, Kang K (2018). Recent progress on multimetal oxide catalysts for the oxygen evolution reaction. Adv. Energy Mater..

[25] Zhu Y (2020). Metal oxide-based materials as an emerging family of hydrogen evolution electrocatalysts. Energy Environ. Sci..

[26] Tahir A (2023). Roles of metal oxide nanostructure-based substrates in sustainable electrochemical water splitting: Recent development and future perspective. ACS Appl. Nano Mater..

[27] Katoch G (2023). Sol-gel auto-combustion developed Nd and Dy co-doped Mg nanoferrites for photocatalytic water treatment, electrocatalytic water splitting and biological applications. J. Water Process Eng..

[28] Kumar S (2023). Synthesis and investigations of structural, surface morphology, electrochemical, and electrical properties of NiFe_2_O_4_ nanoparticles for usage in supercapacitors. J. Mater. Sci. Mater. Electron..

[29] Maitra, S., Mitra, R. & Nath, T. Investigation of electrochemical performance of sol–gel derived MgFe_2_O_4_ nanospheres as aqueous supercapacitor electrode and bi-functional water splitting electrocatalyst in alkaline medium. *Curr. Appl. Phys*. 73–88 (2021).

[30] Jundale VA, Patil DA, Yadav AA (2023). Physical and electrochemical characteristics of NiFe_2_O_4_ thin films as functions of precursor solution concentration. J. Mater. Res..

[31] Hu H (2023). Bifunctional oxygen electrocatalysts enriched with single Fe atoms and NiFe_2_O_4_ nanoparticles for rechargeable zinc–air batteries. Energy Storage Mater..

[32] Benlembarek M, Salhi N, Benrabaa R, Boulahouache A, Trari M (2023). Enhanced photocatalytic performance of NiFe_2_O_4_ nanoparticle spinel for hydrogen production. Int. J. Hydrogen Energy.

[33] Kumar P (2023). Effect of K+ cation doping on structural and morphology of MgFe_2_O_4_ and their role in green electrical energy generation. J. Alloys Compounds.

[34] Mashrah M, Polat S (2023). Hydrothermal synthesis and electrochemical performance of GNPs-doped MgFe_2_O_4_ electrodes for supercapacitors. Solid State Ionics.

[35] Sharifi S, Yazdani A, Rahimi K (2020). Effect of Co_2_^+^ content on supercapacitance properties of hydrothermally synthesized Ni_1__−__x_Co_x_Fe_2_O_4_ nanoparticles. Mater. Sci. Semiconductor Process..

[36] Zong W (2020). Gradient phosphorus-doping engineering and superficial amorphous reconstruction in NiFe_2_O_4_ nanoarrays to enhance the oxygen evolution electrocatalysis. Nanoscale.

[37] Zhang Z, Yan X, Liu J, Liu B, Gu Z-G (2021). Tailoring the catalytic activity of nickel sites in NiFe_2_O_4_ by cobalt substitution for highly enhanced oxygen evolution reaction. Sustain. Energy Fuels.

[38] Wu Z, Wang X, Huang J, Gao F (2018). A Co-doped Ni–Fe mixed oxide mesoporous nanosheet array with low overpotential and high stability towards overall water splitting. J. Mater. Chem. A.

[39] Dang C (2022). A tailored interface engineering strategy designed to enhance the electrocatalytic activity of NiFe_2_O_4_/NiTe heterogeneous structure for advanced energy conversion applications. Mater. Today Nano.

[40] Shinde P, Rout CS, Late D, Tyagi PK, Singh MK (2021). Optimized performance of nickel in crystal-layered arrangement of NiFe_2_O_4_/rGO hybrid for high-performance oxygen evolution reaction. Int. J. Hydrogen Energy.

[41] Israr M, Iqbal J, Arshad A, Gómez-Romero P, Benages R (2020). Multifunctional MgFe_2_O_4_/GNPs nanocomposite: Graphene-promoted visible light driven photocatalytic activity and electrochemical performance of MgFe_2_O_4_ nanoparticles. Solid State Sci..

[42] Xu Y (2019). Supercritical CO_2_-assisted synthesis of NiFe_2_O_4_/vertically-aligned carbon nanotube arrays hybrid as a bifunctional electrocatalyst for efficient overall water splitting. Carbon.

[43] Shombe GB (2022). Tuning composition of CuCo_2_S_4_–NiCo_2_S_4_ solid solutions via solvent-less pyrolysis of molecular precursors for efficient supercapacitance and water splitting. RSC Adv..

[44] Khan MD, Opallo M, Revaprasadu N (2021). Colloidal synthesis of metal chalcogenide nanomaterials from metal–organic precursors and capping ligand effect on electrocatalytic performance: Progress, challenges and future perspectives. Dalton Trans..

[45] Shannon RD (1976). Revised effective ionic radii and systematic studies of interatomic distances in halides and chalcogenides. Acta Crystallogr. Sect. A Crystal Phys. Diffraction Theor. General Crystallogr..

[46] Jacob K, Raj S, Rannesh L (2007). Vegard's law: A fundamental relation or an approximation?. Int. J. Mater. Res..

[47] Naeem M, Shah NA, Gul IH, Maqsood A (2009). Structural, electrical and magnetic characterization of Ni–Mg spinel ferrites. J. Alloys Compounds.

[48] Cullity BD (1956). Elements of X-ray Diffraction.

[49] Dalai N, Mohanty B, Mitra A, Jena B (2019). Highly active ternary Nickel–Iron oxide as bifunctional catalyst for electrochemical water splitting. ChemistrySelect.

[50] Riaz MS (2020). Spherical sacrificial ZnO template-derived hybrid Ni/Co_3_O_4_ cubes as efficient bifunctional electrocatalyst for overall water splitting. Energy Technol..

[51] Jiang J (2019). Fe_2_O_3_ nanocatalysts on N-doped carbon nanomaterial for highly efficient electrochemical hydrogen evolution in alkaline. J. Power Sources.

[52] Zhao Y (2017). Defect-engineered ultrathin δ-MnO_2_ nanosheet arrays as bifunctional electrodes for efficient overall water splitting. Adv. Energy Mater..

[53] Maitra S, Mitra R, Nath T (2021). Investigation of electrochemical performance of sol-gel derived MgFe_2_O_4_ nanospheres as aqueous supercapacitor electrode and bi-functional water splitting electrocatalyst in alkaline medium. Curr. Appl. Phys..

[54] Liu P (2017). P dopants triggered new basal plane active sites and enlarged interlayer spacing in MoS_2_ nanosheets toward electrocatalytic hydrogen evolution. ACS Energy Lett..

[55] Li M (2015). Facile synthesis of electrospun MFe_2_O_4_ (M = Co, Ni, Cu, Mn) spinel nanofibers with excellent electrocatalytic properties for oxygen evolution and hydrogen peroxide reduction. Nanoscale.

[56] Hirai S (2016). Enhancement of the oxygen evolution reaction in Mn_3_^+^-based electrocatalysts: Correlation between Jahn-Teller distortion and catalytic activity. RSC Adv..

[57] Chen Z, Kronawitter CX, Koel BE (2015). Facet-dependent activity and stability of Co_3_O_4_ nanocrystals towards the oxygen evolution reaction. Phys. Chem. Chem. Phys..

