# Supplementary material for: Eco-friendly mixed metal (Mg–Ni) ferrite nanosheets for efficient electrocatalytic water splitting
Source: Sci Rep. 2023 Dec 13;13:22179. doi: 10.1038/s41598-023-49259-y (PMC10719248; doi:10.1038/s41598-023-49259-y)
Supplement: Supplementary file 1 — Supplementary Information. [file 41598_2023_49259_MOESM1_ESM.docx]

Supporting Information

**Eco-friendly mixed metal (Mg-Ni) ferrite nanosheets for efficient electrocatalytic water splitting**

Nyemaga M. Malima,^1,2^ Malik Dilshad Khan,^1^* Siphamandla Masikane,^1^ Felipe M. de Souza,^3^ Jonghyun Choi,^3^ Ram K. Gupta^3^ and Neerish Revaprasadu^1^*

*^1^Department of Chemistry, University of Zululand, Private Bag X1001, KwaDlangezwa 3880, South Africa.*

*^2^Department of Chemistry, College of Natural and Mathematical Sciences, University of Dodoma, P.O. Box 338, Dodoma, Tanzania.*

*^3^Department of Chemistry, National Institute for Materials Advancement, Pittsburg State University, Pittsburg, Kansas 66762, United States.*

***Email**: [RevaprasaduN@unizulu.ac.za](mailto:RevaprasaduN@unizulu.ac.za); [malikdilshad@hotmail.com](mailto:malikdilshad@hotmail.com)


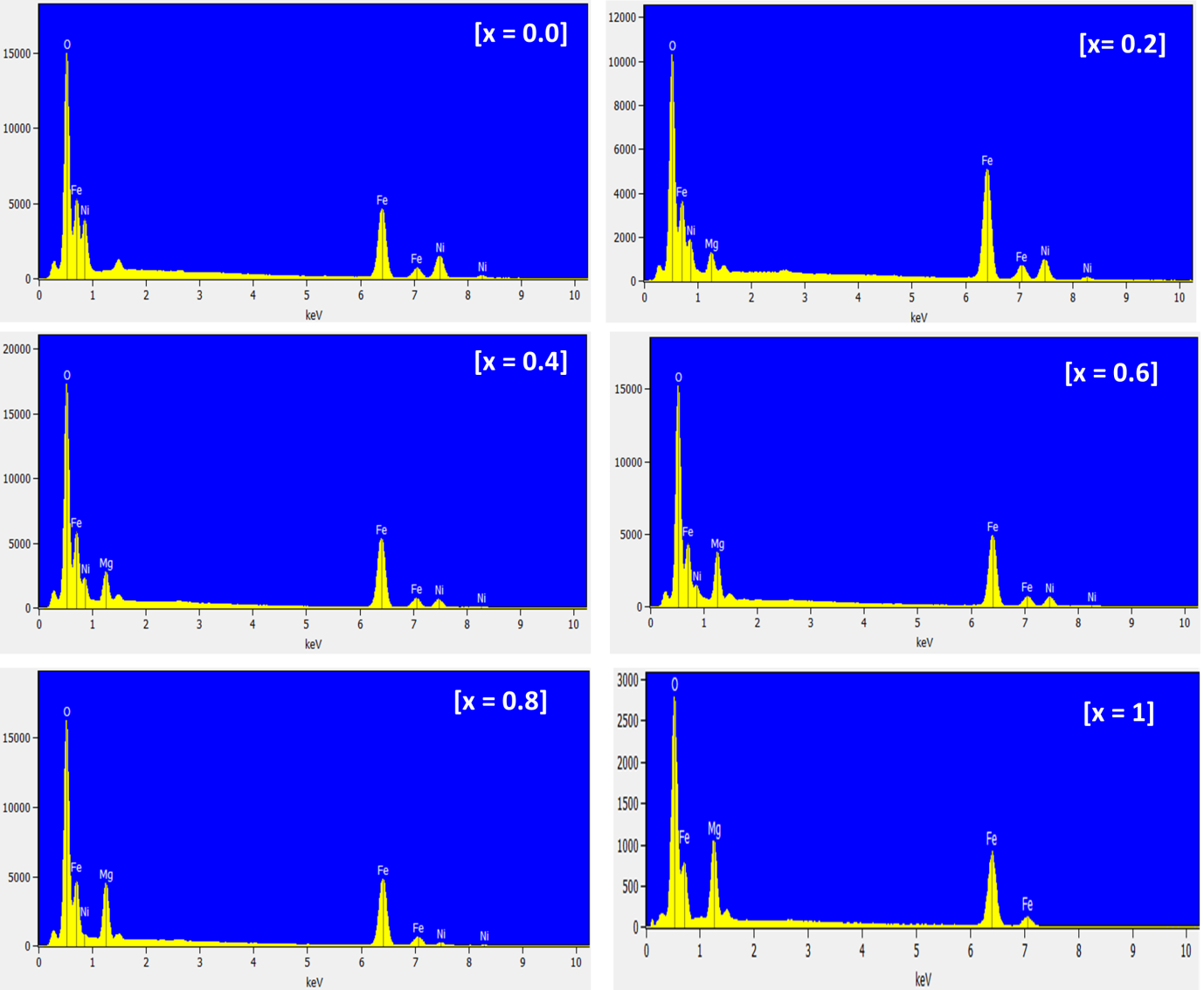


**Figure S1.** EDX spectra of Ni_1-x_Mg_x_Fe_2_O_4_ (0 ≤ x ≤ 1) nanoparticles.


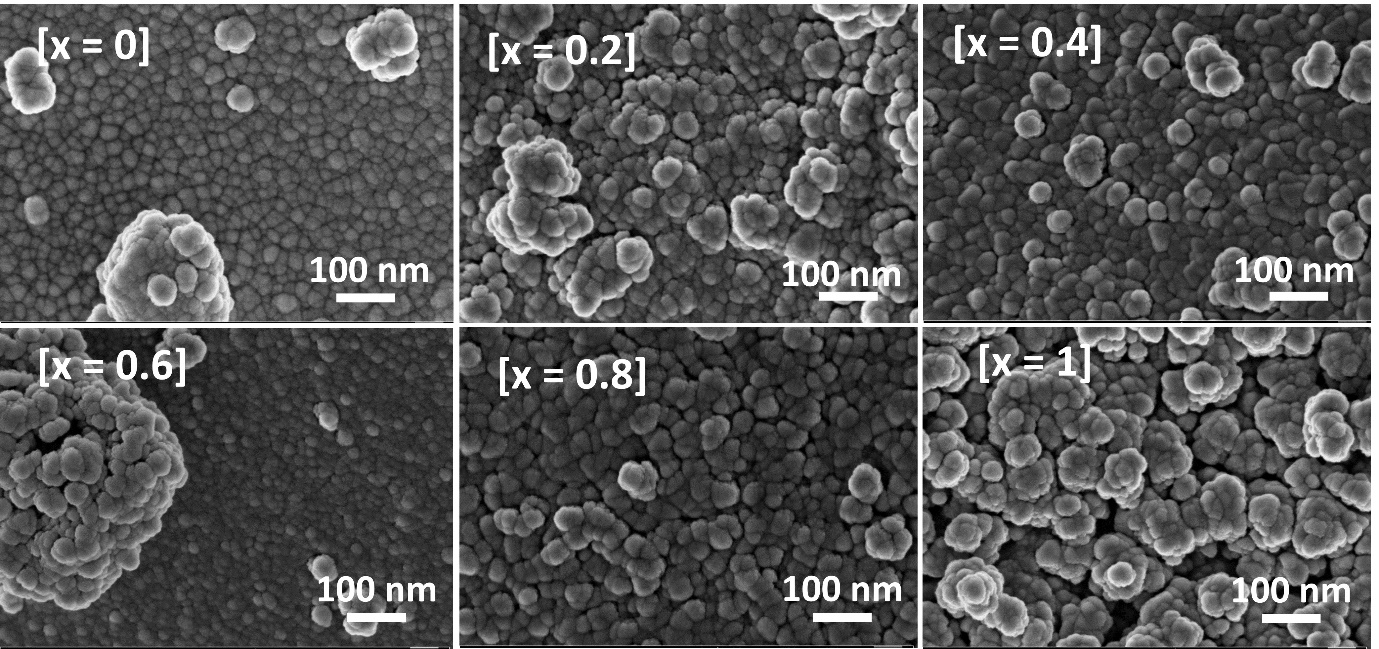


**Figure S2.** SEM images of Ni_1-x_Mg_x_Fe_2_O_4_ solid solutions over the entire range (scale bar = 100 ).


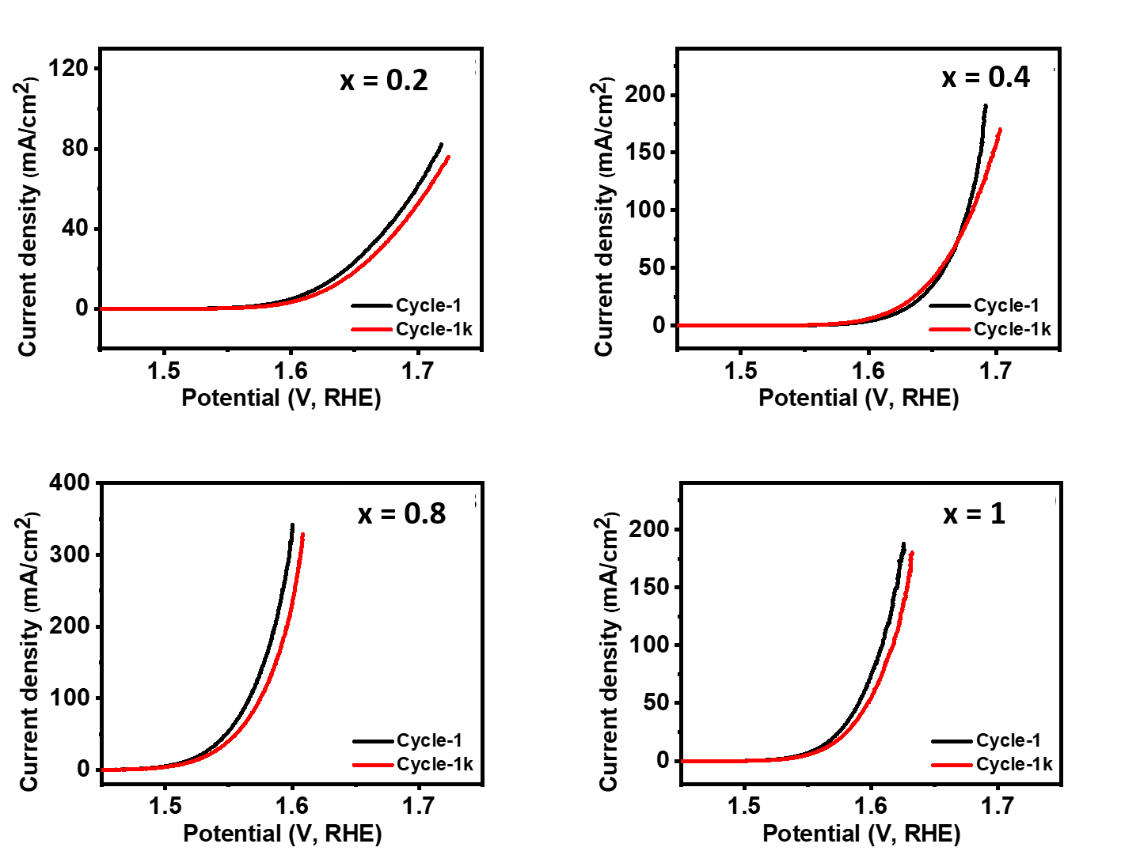


**Figure S3.** HER polarization curves at various cycles for the various samples.


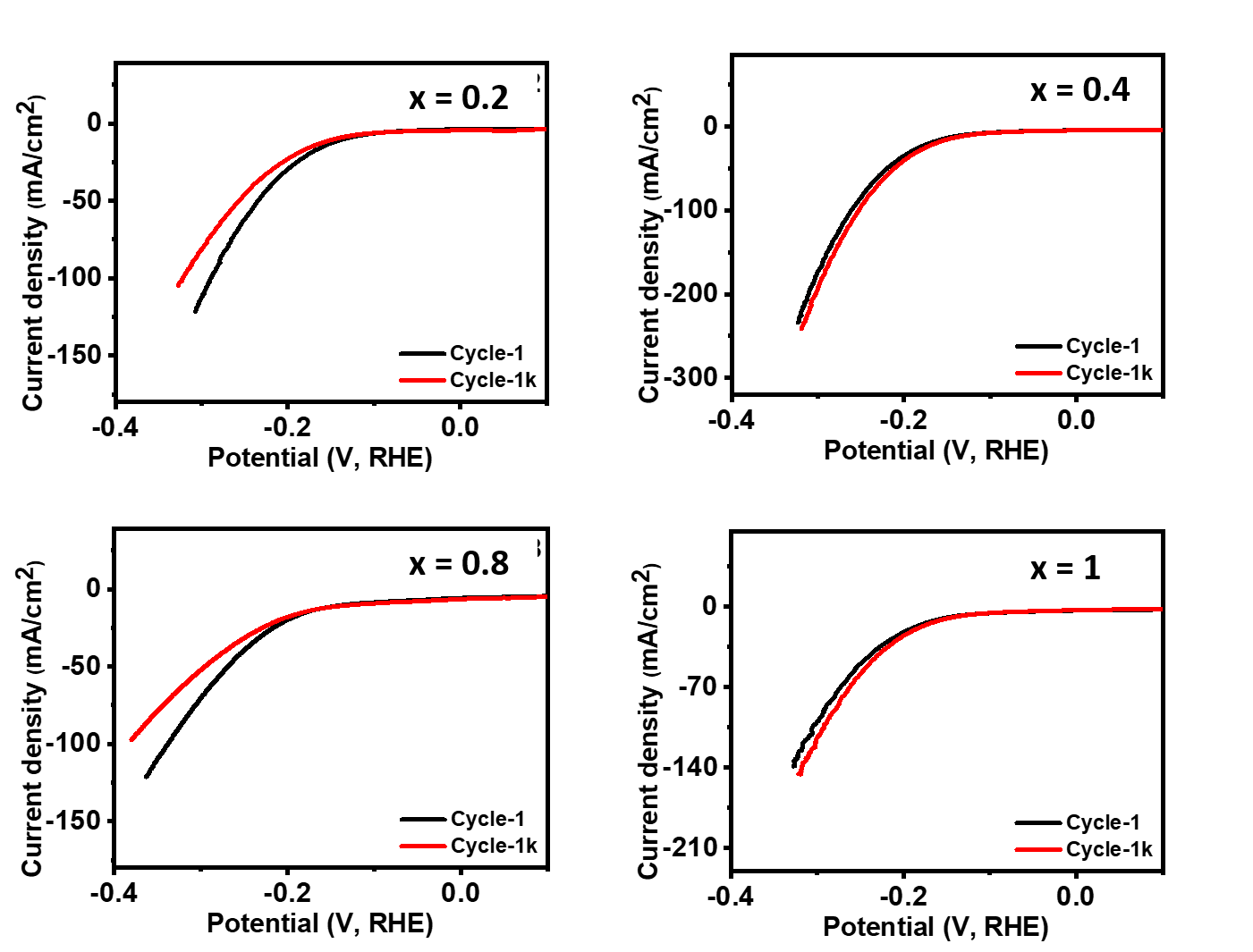


**Figure S4.** OER polarization curves at various cycles for the various samples.

**Table S1**. Summary of the theoretical and experimental atomic % for Ni_1-x_Mg_x_Fe_2_O_4_ solid solutions obtained from EDX analysis.

| **(x)** | | **Theoretical atomic %** | | | | | **Atomic % from EDX** | | | | |
| --- | --- | --- | --- | --- | --- | --- | --- | --- | --- | --- | --- |
|  | **Ni** | | **Mg** | **Fe** | **O** |  | **Ni** | **Mg** | **Fe** | **O** |  |
| 0 | 14.3 | | - | 28.6 | 57.1 |  | 14.6 | - | 25.9 | 59.6 |  |
| 0.2 | 11.4 | | 2.9 | 28.6 | 57.1 |  | 10.4 | 3.3 | 28.2 | 58.2 |  |
| 0.4 | 8.6 | | 5.7 | 28.6 | 57.1 |  | 6.6 | 5.7 | 29.0 | 59.1 |  |
| 0.6 | 5.7 | | 8.6 | 28.6 | 57.1 |  | 5.8 | 8.3 | 27.2 | 58.7 |  |
| 0.8 | 2.9 | | 11.4 | 28.6 | 57.1 |  | 1.8 | 10.7 | 26.8 | 60.7 |  |
| 1 | - | | 14.3 | 28.6 | 57.1 |  | - | 14.1 | 26.1 | 59.8 |  |

**Table S2.** Comparison of HER performance of Ni_0.4_Mg_0.6_Fe_2_O_4_ with previously reported electrocatalysts in alkaline electrolyte.

| **Catalyst** | **Preparation method** | \| **η_10_ (mV) in 1 M KOH** \| \| --- \| | **Tafel slope**  **(mV/dec)** | **Reference** |
| --- | --- | --- | --- | --- | --- |
| MgFe_2_O_4_ | Sol-gel | 402 | 241 | ^1^ |
| N-doped Ni_3_S_2_/VS_2_ | Hydrothermal | 151 | 107.5 | ^2^ |
| Ni-MoSe_2_ | Hydrothermal | 206 | 81 | ^3^ |
| CuO@Cu_2_O | Solid state reaction | 135 | 135 | ^4^ |
| MoSe_2_ | Solvothermal | 310 | 93 | ^5^ |
| Ni_3_S_2_ | Chemical vapor deposition | 116 | 96 | ^6^ |
| NiS | Chemical vapor deposition | 124 | 154 | ^6^ |
| MoP/MoNiP@C | Calcination and phosphorization | 134 | 66 | ^7^ |
| S_2_-NiCoP_x_/NF | Hydrothermal, phosphorization and sulfuration | 144 | 66 | ^8^ |
| CuFe_2_O_4_ | Ball milling | 241(6 M KOH) | 114 | ^9^ |
| FeSe_2_/CoFe_2_O_4_ | Hydrothermal | 231 | 88.76 | ^10^ |
| NiS/Ni_3_S_4_ | Hydrothermal | 221 | - | ^11^ |
| Fe-doped α-NiS | Solventless | 146 | 113 | ^12^ |
| CoSe_2_/MoSe_2_ | Solvothermal | 218 | 76 | ^13^ |
| Cu-doped NiS | Solventless | 154 | 114 | ^12^ |
| Co-doped NiS | Solventless | 156 | 98 | ^12^ |
| Fe_2_O_3_ |  | 376 | 173 | ^14^ |
| Ni-NiO |  | 516 | 207 | ^14^ |
| Ni-Ni Fe_2_O_4_ | Hydrothermal  followed by thermal decomposition | 217 | 96 | ^14^ |
| NiFe_2_O_4_ | Hydrothermal | 187 | 122.7 | ^15^ |
| Exfoliated NiFe_2_O_4_ | Hydrothermal | 274 | 85.8 | ^15^ |
| NiFe_2_O_4_@N/rGO -800 °C | Pyrolytic method | 157 | 94.7 | ^16^ |
| NiFe_2_O_4_@rGO - 800 °C | Pyrolytic method | 186 | 129.9 | ^16^ |
| **Ni_0.4_Mg_0.6_Fe_2_O_4_** | **Solventless** | **121** | **125** | **This work** |

**Table S3.** Comparison of OER performance of Ni_0.2_Mg_0.8_Fe_2_O_4_ with previously reported electrocatalysts in alkaline electrolyte.

| **Catalyst** | **Preparation method** | \| **η_10_ (mV)** \| \| --- \|   **in 1 M KOH** | **Tafel slope**  **(mV/decade)** | **Reference** |
| --- | --- | --- | --- | --- | --- |
| MgFe_2_O_4_ | Sol-gel | 1090 | 317 | ^1^ |
| LiCoO_2_ |  | 520 | 48 | ^17^ |
| CoCr_2_O_4_ | Calcination | 422 | 63.3 | ^18^ |
| Ni_59_Cu_19_P_9_ | Electrodeposition | 307 | 42.5 | ^19^ |
| FeC/MnO_2_ | Wet-chemical | 254 | 39.81 | ^20^ |
| MnO_2_ | Wet-chemical | 364 | 60.84 | ^20^ |
| CuO@Cu_2_O | Solid state reaction | 315 | 63 | ^4^ |
| CeO_2_/NiFeCo | Precipitation | 260 | - | ^21^ |
| Co_3_S_4_ | Solvothermal | 310 | 84.7 | ^22^ |
| Ni_5_P_4_ | Hot injection | 350 (50 mA/cm^2^) | - | ^23^ |
| NiCoS | Solvothermal [sulfidation](https://www.sciencedirect.com/topics/engineering/sulfidation)  and [calcination](https://www.sciencedirect.com/topics/chemistry/calcination) | 320 | 58.8 | ^24^ |
| CrMnFeCoNi)S*_x_* | Pulse thermal decomposition | 295 (100 mA/cm^2^) | 66 | ^25^ |
| FeCo_2_S_4_NTA/CC | Template-free | 317 (100 mA/cm^2^) | 36 | ^26^ |
| V-doped NiS_2_ | Hydrothermal | 290 | 90 | ^27^ |
| Fe_2_O_3_ | Hydrothermal followed by decomposition | 304 | 84 | ^14^ |
| Ni-NiO | Hydrothermal followed by decomposition | 397 | 104 | ^14^ |
| S-NiO | Electrodeposition and calcination | 306 | 148 | ^28^ |
| S-NiFe_2_O_4_ | Thiourea-assisted [electrodeposition](https://www.sciencedirect.com/topics/materials-science/electrodeposition) and calcination | 343 | 124.5 | ^28^ |
| NiFe_2_O_4_ | Hydrothermal followed by decomposition | 342 | 44 | ^29^ |
| NiO | Hydrothermal followed by decomposition | 364 | 58 | ^29^ |
| NiO-Ni Fe_2_O_4_/rGO | Hydrothermal–calcination method | 296 | 42.8 | ^30^ |
| MgFe_2_O_4_ | Sol-gel | 1090 | 317 | ^1^ |
| NiO/NiFe_2_O_4_ | Hydrothermal followed by decomposition | 302 | 42 | ^29^ |
| **Ni_0.2_Mg_0.8_Fe_2_O_4_** | **Solventless** | **284** | **58** | **This work** |

**References**

1 Maitra, S., Mitra, R. & Nath, T. Investigation of electrochemical performance of sol-gel derived MgFe2O4 nanospheres as aqueous supercapacitor electrode and bi-functional water splitting electrocatalyst in alkaline medium. *Current Applied Physics* **27**, 73-88 (2021).

2 Zhong, X. *et al.* 3D heterostructured pure and N-Doped Ni3S2/VS2 nanosheets for high efficient overall water splitting. *Electrochimica Acta* **269**, 55-61 (2018).

3 Zhao, G. *et al.* Heteroatom‐doped MoSe2 Nanosheets with Enhanced Hydrogen Evolution Kinetics for Alkaline Water Splitting. *Chemistry–An Asian Journal* **14**, 301-306 (2019).

4 Xing-Xing, M., Li, C., ZHANG, Z. & Ji-Lin, T. Electrochemical Performance Evaluation of CuO@ Cu2O Nanowires Array on Cu Foam as Bifunctional Electrocatalyst for Efficient Water Splitting. *Chinese Journal of Analytical Chemistry* **48**, e20001-e20012 (2020).

5 Meiron, O. E., Kuraganti, V., Hod, I., Bar-Ziv, R. & Bar-Sadan, M. Improved catalytic activity of Mo 1− x W x Se 2 alloy nanoflowers promotes efficient hydrogen evolution reaction in both acidic and alkaline aqueous solutions. *Nanoscale* **9**, 13998-14005 (2017).

6 Kajbafvala, M., Moradlou, O. & Moshfegh, A. Z. CVD growth of the nanostructured Ni3S2 thin films as efficient electrocatalyst for hydrogen evolution reaction. *Vacuum* **188**, 110209 (2021).

7 Li, J. *et al.* Bimetallic phosphides as high-efficient electrocatalysts for hydrogen generation. *Inorganic Chemistry* **60**, 1624-1630 (2021).

8 Sun, Q. *et al.* Dual anions engineering on nickel cobalt-based catalyst for optimal hydrogen evolution electrocatalysis. *Journal of Colloid and Interface Science* **589**, 127-134 (2021).

9 Tan, J., Xu, S., Zhang, H., Cao, H. & Zheng, G. Preparation of a porous bulk copper ferrite spinel with high performance in the electrolysis of water. *Electrochimica Acta* **381**, 138199 (2021).

10 Zhang, H. *et al.* Successfully synthesis of FeSe2/CoFe2O4 heterojunction with high performance for hydrogen evolution reaction. *Renewable Energy* **155**, 717-724 (2020).

11 Qin, Z. *et al.* Composition-dependent catalytic activities of noble-metal-free NiS/Ni3S4 for hydrogen evolution reaction. *The Journal of Physical Chemistry C* **120**, 14581-14589 (2016).

12 Shombe, G. B. *et al.* Unusual doping induced phase transitions in NiS via solventless synthesis enabling superior bifunctional electrocatalytic activity. *Sustainable Energy & Fuels* **4**, 5132-5143 (2020).

13 Zhao, G. *et al.* CoSe2/MoSe2 heterostructures with enriched water adsorption/dissociation sites towards enhanced alkaline hydrogen evolution reaction. *Chemistry–A European Journal* **24**, 11158-11165 (2018).

14 Zhang, J. *et al.* Ultrathin carbon coated mesoporous Ni-NiFe2O4 nanosheet arrays for efficient overall water splitting. *Electrochimica Acta* **321**, 134652 (2019).

15 Munonde, T. S. *et al.* A green approach for enhancing the electrocatalytic activity and stability of NiFe2O4/CB nanospheres towards hydrogen production. *Renewable Energy* **154**, 704-714 (2020).

16 Cao, L., Li, Z., Su, K., Zhang, M. & Cheng, B. Rational design of hollow oxygen deficiency-enriched NiFe2O4@ N/rGO as bifunctional electrocatalysts for overall water splitting. *Journal of Energy Chemistry* **54**, 595-603 (2021).

17 Lu, Z. *et al.* Electrochemical tuning of layered lithium transition metal oxides for improvement of oxygen evolution reaction. *Nature communications* **5**, 1-7 (2014).

18 Al‐Mamun, M. *et al.* Strongly coupled CoCr2O4/carbon nanosheets as high performance electrocatalysts for oxygen evolution reaction. *Small* **12**, 2866-2871 (2016).

19 Kim, B. K., Kim, S.-K., Cho, S. K. & Kim, J. J. Enhanced catalytic activity of electrodeposited Ni-Cu-P toward oxygen evolution reaction. *Applied Catalysis B: Environmental* **237**, 409-415 (2018).

20 Ye, M. *et al.* Hierarchical FeC/MnO2 composite with in-situ grown CNTs as an advanced trifunctional catalyst for water splitting and Metal− Air batteries. *Ceramics International* **47**, 18424-18432 (2021).

21 Wang, Y. & Gao, F.-m. Efficient fish-scale CeO2/NiFeCo composite material as Electrocatalyst for oxygen evolution reaction. *Nanotechnology* (2021).

22 Zhu, M. *et al.* Hydrophilic cobalt sulfide nanosheets as a bifunctional catalyst for oxygen and hydrogen evolution in electrolysis of alkaline aqueous solution. *Journal of colloid and interface science* **509**, 522-528 (2018).

23 Ayom, G. E. *et al.* Flexible molecular precursors for selective decomposition to nickel sulfide or nickel phosphide for water splitting and supercapacitance. *Chemistry–A European Journal* **26**, 2693-2704 (2020).

24 Yu, Z. *et al.* MOF-directed templating synthesis of hollow nickel-cobalt sulfide with enhanced electrocatalytic activity for oxygen evolution. *International Journal of Hydrogen Energy* **43**, 8815-8823 (2018).

25 Cui, M. *et al.* High‐Entropy Metal Sulfide Nanoparticles Promise High‐Performance Oxygen Evolution Reaction. *Advanced Energy Materials* **11**, 2002887 (2021).

26 Hu, X., Wang, R., Sun, P., Xiang, Z. & Wang, X. Tip-welded ternary FeCo2S4 nanotube arrays on carbon cloth as binder-free electrocatalysts for highly efficient oxygen evolution. *ACS Sustainable Chemistry & Engineering* **7**, 19426-19433 (2019).

27 Liu, H. *et al.* Electronic structure reconfiguration toward pyrite NiS2 via engineered heteroatom defect boosting overall water splitting. *ACS nano* **11**, 11574-11583 (2017).

28 Liu, J., Zhu, D., Ling, T., Vasileff, A. & Qiao, S.-Z. S-NiFe2O4 ultra-small nanoparticle built nanosheets for efficient water splitting in alkaline and neutral pH. *Nano Energy* **40**, 264-273 (2017).

29 Liu, G., Gao, X., Wang, K., He, D. & Li, J. Uniformly mesoporous NiO/NiFe2O4 biphasic nanorods as efficient oxygen evolving catalyst for water splitting. *International Journal of Hydrogen Energy* **41**, 17976-17986 (2016).

30 Zhang, G., Li, Y., Zhou, Y. & Yang, F. NiFe Layered‐Double‐Hydroxide‐Derived NiO‐NiFe2O4/Reduced Graphene Oxide Architectures for Enhanced Electrocatalysis of Alkaline Water Splitting. *ChemElectroChem* **3**, 1927-1936 (2016).
